# Tunable white light emission by variation of composition and defects of electrospun Al_2_O_3_–SiO_2_ nanofibers

**DOI:** 10.3762/bjnano.6.29

**Published:** 2015-01-28

**Authors:** Jinyuan Zhou, Gengzhi Sun, Hao Zhao, Xiaojun Pan, Zhenxing Zhang, Yujun Fu, Yanzhe Mao, Erqing Xie

**Affiliations:** 1 School of Physical Science and Technology, Lanzhou University, Lanzhou, Gansu 730000, People’s Republic of China,; 2 School of Mechanical and Aerospace Engineering, Nanyang Technological University, 50 Nanyang Avenue, 639798, Singapore

**Keywords:** Al_2_O_3_–SiO_2_, defects, electrospinning, nanofibers, photoluminescence, white light emission

## Abstract

Composite nanofibers consisting of Al_2_O_3_–SiO_2_ were prepared by electrospinning in combination with post-calcination in air. X-ray diffraction, scanning electron microscopy, and transmission electron microscopy were used to investigate the crystalline phase and microstructure of the composite nanofibers. Photoluminescence experiments indicated that the resulting white light emission can be tuned by the relative intensity of the individual spectral components, which are related to the individual defects such as: violet-blue emission from O defects, green emission from ≡Si(Al)–O–C∙=O, and red emission from intersystem radiative crossing. White light emission was realized at a Al/(Al–Si) ratio of 40 and 60 mol %. This research may offer a deeper understanding of the preparation of efficient and environmentally friendly, white luminescence materials.

## Introduction

During the last decade, nanoscale SiO_2_ has been intensely investigated as a new silicon-based light-emitting material. Its wide photoluminescence (PL) band ranges from the UV to red wavelengths, allowing for potential application in white light emission devices. It has been demonstrated that the luminescence emission and emission intensity of SiO_2_ nanostructures are strongly dependent upon the intrinsic structural defects and extrinsic environmental influences introduced during the preparation processes, which can be effectively tuned and controlled by doping [1–4]. Thus far, in order to achieve enhanced and stable light emission, various materials have been incorporated into a SiO_2_ matrix, such as Si nanocrystals, carbon nanocomposites, ZnO, Al_2_O_3_, SnO_2_, and various rare-earth elements [5–9].

Among those materials, Al_2_O_3_ is one of the most important materials in the history of ceramics, and has been extensively applied in catalysts, coatings, microelectronics and various devices, due to its excellent physical and chemical stability, high dielectric constant, wide band gap energy, and relatively high refractive index [10]. Similar to SiO_2_, Al_2_O_3_ is inexpensive and environmentally friendly, as well as highly compatible with the current integrated circuit processes. It has been demonstrated that the PL properties of SiO_2_/Al_2_O_3_ composites are more suitable than those of pure SiO_2_ or Al_2_O_3_ [11–15]. For example, Hayakawa et al. reported on the PL properties of 10Al_2_O_3_–90SiO_2_ glasses annealed at 500 °C, and found two emission peaks at 420 and 520 nm, which are assigned to the point defects of oxygen deficiencies and the radical carbonyl defect (≡Si(Al)–O–C∙=O) formed on the pore surface [16]. Mir et al. incorporated 30 nm, Al_2_O_3_ nanocrystals into silica aerogels, followed by calcination at 1150 °C in air for 2 h. The resulting 1Al_2_O_3_–3SiO_2_ composites exhibited strong, visible PL bands ranging from 400 to 600 nm centered at ≈500 nm, which were assigned to OH-related radiative emission centers formed in the samples [11]. Additionally, Yoldas also showed that the A1_2_O_3_–SiO_2_ composites respond to UV light by emission of strong, visible luminescence (400–700 nm), which is due to the (≡Si–O¨O–Si≡) radiative centers [17]. Chen et al. reported a peapod-like heterostructure composed of SiO*_x_* particles orderly embedded in the highly crystalline α-Al_2_O_3_ nanoribbons. They observed a strong and stable blue emission centered at 467 nm under excitation at 320 nm, which was attributed to the neutral oxygen vacancies (≡Si–Si≡) in the SiO*_x_*–Al_2_O_3_ heterostructure [18]. More recently, Korsunska et al. have investigated the PL behaviors of Si-rich Al_2_O_3_ films annealed at 1150 °C and observed intense emission in the visible spectral range from 575 to 600 nm, which is ascribed to defects in the matrix located near the nanocrystal/matrix interface [13–15,19]. From the above referenced work, it can be seen that the mechanism of this defect-dominant PL still remains ambiguous, and it is also a challenge to obtain the desired white luminescent material by control the different defects. Moreover, to date, few studies have reported on Al_2_O_3_–SiO_2_ nanocomposites. Thus, it is important and instructive to further explore the preparation and PL properties of these environmentally friendly, Al_2_O_3_–SiO_2_ nanomaterials.

In this work, Al_2_O_3_–SiO_2_ composite nanofibers with different Al/(Al–Si) ratios were prepared by electrospinning in combination with calcination in air. Strong light emission was observed from the Al_2_O_3_–SiO_2_ hetero-nanofibers with tunable emission from bluish-white to yellow-white. The possible origins of each PL band in this composite nanofiber were also discussed.

## Results and Discussion

### Crystalline structures

**Figure 1:**
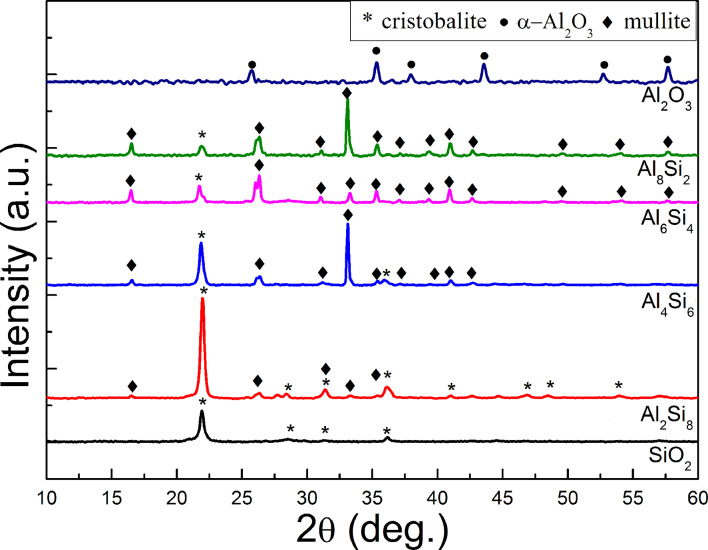
XRD patterns of the obtained composite nanofibers with different Al/(Al–Si) ratios.

Figure 1 shows the XRD patterns of the samples with different Al/(Al–Si) ratios annealed at 1200 °C in air. The diffraction peaks from pure SiO_2_ are located at 21.9°, 28.5°, and 36.2°, which can be assigned to the <101>, <111>, and <200> crystalline plane of cristobalite (ICDD No. 39-1425), respectively [20–21]. The diffraction peaks of pure Al_2_O_3_ are located at 25.4°, 34.92° , 43.16°, 52.36° , and 57.30°,which can be assigned to the <012>, <104>, <110>, <113>, <024>, and <110> crystalline plane of α-Al_2_O_3_ (ICDD No. 46-1212), respectively [22]. Once the Al_2_O_3_ components are mixed with SiO_2_, the mullite formation reaction in diphasic gels takes place between amorphous silica and transition alumina during calcination. It can be seen from the typical mullite XRD line from Si_6_Al_4_ samples that the diffraction peaks located at 16.51°, 26.25°, 31.05°, 33.23°, 35.27°, 37.05°, 39.25°, 40.86°, 42.65°, 49.55°, 54.09°, and 57.58° can be assigned to the <100>, <210>, <001>, <220>, <111>, <130>, <201>, <121>, <230>, <311>, <321>, and <041> crystalline planes of mullite (ICDD No. 15-0776), respectively [23–24]. It is also noted that the diffraction peaks from mullite located at 26.25° are clearly split into two peaks, <120> and <210>, which is due to the fast heating process during the calcination of Al_2_O_3_–SiO_2_ gels [25].

Furthermore, a diffraction peak is located at approximately 22°, corresponding to the <101> of cristobalite, which indicates that crystalline silica phases are present in the diphasic gels during heating, while α-Al_2_O_3_ phases can barely be detected. Wei et al. found that A1_2_O_3_ or cristobalite residue is formed in a Al_2_O_3_-rich or SO_2_-rich, mullite specimen if the A1_2_O_3_ content of the sample is not maintained between 60 and 66 mol % [26]. In our case, the mole percent of Al_2_O_3_ in the diphasic gels ranges from 11 mol % to 66 mol %, that is, most of our diphasic gels are silica-rich, except Al_8_Si_2_. The cristobalite residues in the Al-rich Al_8_Si_2_ samples might be caused by Si contamination from the silicon substrates used during high-temperature calcination.

### Morphology and microstructure

**Figure 2:**
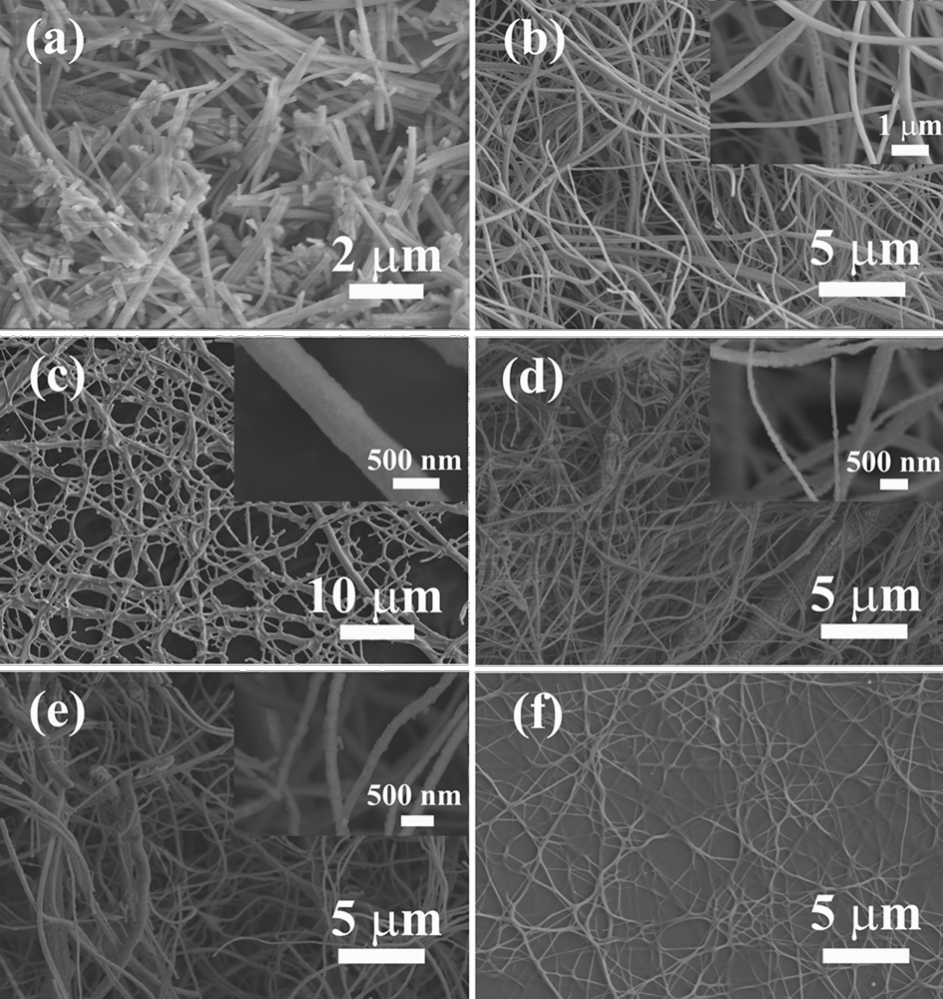
SEM images of the obtained composite nanofibers with different Al/(Al–Si) ratios: (a) Al_2_O_3_, (b) Al_8_Si_2_, (c) Al_6_Si_4_, (d) Al_4_Si_6_, (e) Al_2_Si_8_, and (f) SiO_2_. The insets are their corresponding enlarged SEM images.

Figure 2a illustrates the morphology of the pure Al_2_O_3_ nanofibers. The diameter of the rather brittle fibers is about 100–200 nm, exhibiting a smooth surface. When 20 mol % SiO_2_ is incorporated into the Al_2_O_3_ matrix, the fibers become ductile with length up to the centimeter scale and a diameter similar to the pure Al_2_O_3_ material. Moreover, from the enlarged SEM image shown in the inset of Figure 2b, some black spots were formed on the surface of fibers. This may be due to the precipitation of mullite nanocrystals from the inside to the surface of the Al_2_O_3_ during the calcination [18]. When the concentration of SiO_2_ is further increase to 40 mol % (Figure 2c), the composite fibers show an obvious change, exhibiting a fused, interconnect network with a diameter of ≈500 nm. This may be caused by the formation of mullite components in the samples. The continued increase in the concentration of SiO_2_ (to 60 and 80 mol %, as shown in Figure 2d and Figure 2e, respectively) results in the coarsening of the surface of the fiber, further implying the precipitation of mullite nanocrystals from the inside to the surface of the fibers during the calcination. Comparably, the pure SiO_2_ nanofiber has a diameter of ≈100 nm with smooth surface (Figure 2f).

**Figure 3:**
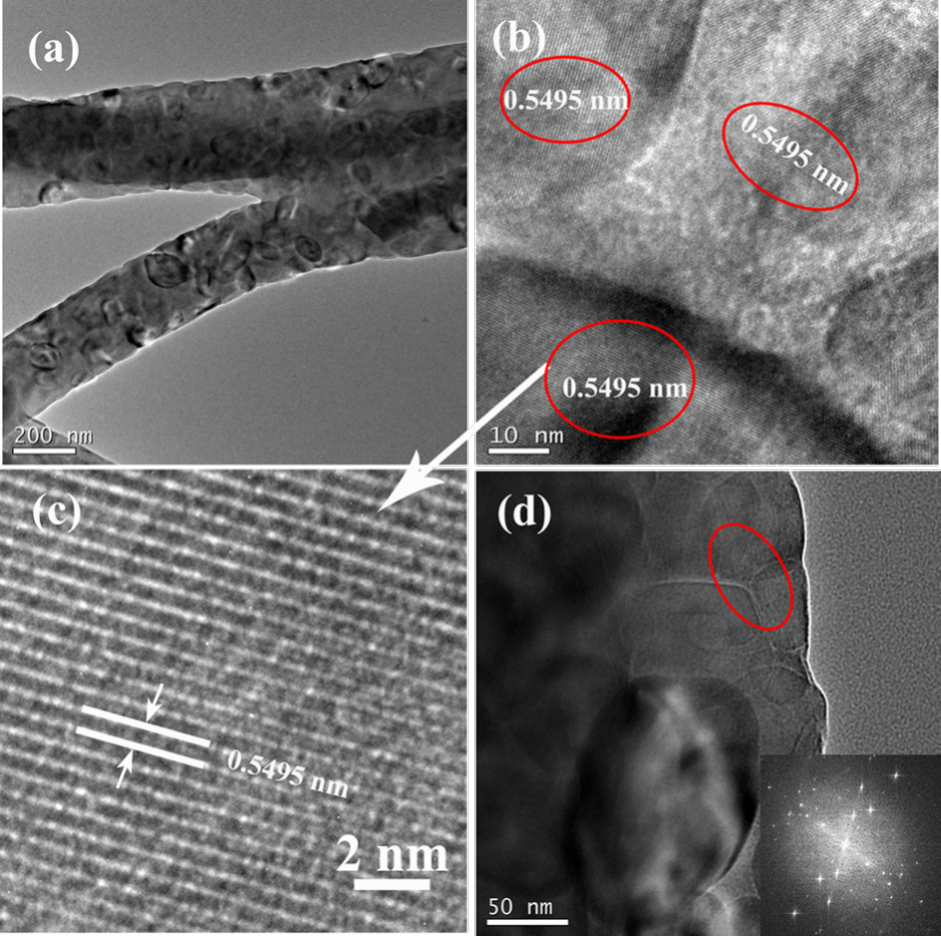
(a) Low magnification TEM image of Al_4_Si_6_ nanofibers; (b) locally enlarged TEM image; (c) HRTEM image of one area shown in (b); and (d) enlarged TEM image of the fiber surface, the inset is the SAED pattern collected from the fiber’s edge.

Further studies on the microstructure and morphology of the calcined composite nanofibers were conducted by TEM. Figure 3a shows the morphology of the Al_4_Si_6_ fibers. It can be seen that the fibers have diameters of about 100–200 nm with a coarse surface, which is consistent with the above SEM results. Additionally, many nanocrystals can be observed in the enlarged TEM image shown in Figure 3b with dimensions from several nm to several tens of nm. The HRTEM image in Figure 3c illustrates that the lattice fringes are well-defined, suggesting that the composite nanowires have a high degree of crystallinity. The interplanar spacing of 0.5495 nm measured from the legible lattice fringes along the axis of the nanowire is quite similar to that of the <110> planes of the mullite crystals [27–28]. In addition, some nanocrystals of cristobalite with dimensions of several tens of nm can also found on the surface of the fibers, as shown in Figure 3d. Selected area electron diffraction (SAED) patterns are collected from the thin edge of one fiber, as shown in the inset of Figure 3c. The patterns not only verify the high degree of crystallinity of the composite nanofibers, but also indicate the disordered stacking of the formed mullite nanocrystals.

### Chemical bonds

**Figure 4:**
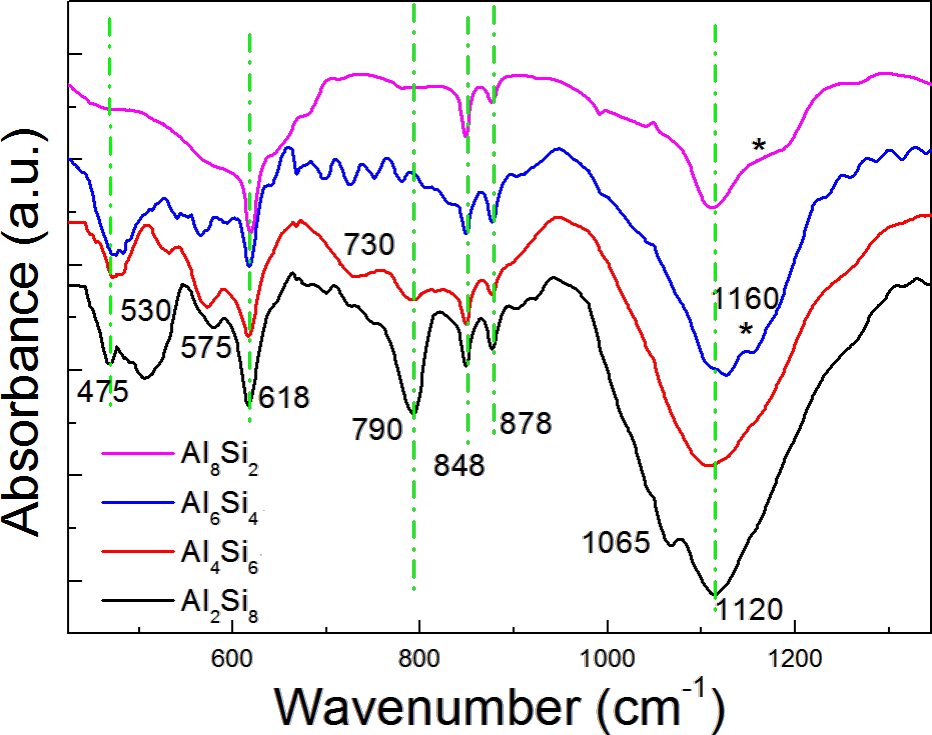
FTIR spectra of the composite nanofibers with different Al–Si ratios (400–1320 cm^−1^).

Previous results indicate the PL from Al_2_O_3_–SiO_2_ composites is mainly due to various types of defects formed during calcination in air. Thus, to investigate the chemical bonds in the samples, FTIR measurements were also conducted. Selected IR spectra of the samples with different Al/(Al–Si) ratios are shown in Figure 4, and the corresponding assignments are listed in Table 1. The IR spectra of our samples are similar to those reported for natural and synthetic silica/alumina composites [29–37]. In the wavenumber range of 400–1300 cm^−1^, nine, obvious, characteristic, IR peaks from the composite samples are observed and are located at 475, 530, 573, 618, 730, 790, 848, 878, and 1120 cm^−1^. The IR peak at ≈475 cm^−1^ is due to the vibrations of O–Si–O bending modes (SiO_4_), whose intensity increases with an increase in the Si content. Typically, the stretching modes of an AlO_6_ moiety are expected in the region 500–680 cm^−1^, whilst comparable modes for AlO_4_ appear in the region of 680–880 cm^−1^ [29–30]. Thus, the characteristic, broad adsorption peaks at 530, 575, and 618 cm^−1^ are assigned to Al–O stretching modes (AlO_6_), and the peaks at 730, 848, and 878 cm^−1^ are due to the vibrations of Al–O stretching modes (AlO_4_) [31–32]. It can be seen that the IR peak at 790 cm^−1^ increases with increasing the Si content, indicating that this peak is related to the Si components. Referring to previous literature [33–34], this IR peak at 790 cm^−1^ likely corresponds to the vibration of Si–C–O bonds formed due to the residual carbon elements from PVP or ethanol.

**Table 1:** FTIR absorbance band assignments in the region of 400–1320 cm^−1^ for Al_2_O_3_–SiO_2_ composite nanofibers.

band notation	band position (cm^−1^)	band assignment

1	472, 1120	O–Si–O bending (SiO_4_)
2	507, 530, 575, 618	Al–O stretching (AlO_6_)
3	730, 848, 878, 1160	Al–O stretching (AlO_4_)
4	790	Si–C–O bands
5	1065	SiO–CO

In the range of 950–1330 cm^−1^, the main peak intensity increases with increasing Si content, and this peak at ≈1120 cm^−1^ should be assigned to the vibrations of Si–O–Si stretching modes (SiO_4_). Moreover, it is seen that the Si–O–Si stretching vibration broadens with increasing Al content, which is due to the formation of Al–O–Si bonds [35]. The absorption peak from the samples with high Al content can be split into two peaks at ≈1120 cm^−1^ and 1160 cm^−1^, indicating a high content of mullite [29], which is consistent with our XRD results. The new split IR peak at 1160 cm^−1^ is due to Al–O stretching modes (AlO_4_) [32]. In addition, from the FTIR spectrum of Si_8_Al_2_ samples, the sharp, shallow absorption peak, appearing at 1065 cm^−1^, corresponds to asymmetric stretching of Si–O–Si or Si–O– defects [29].

### Photoluminescence properties

**Figure 5:**
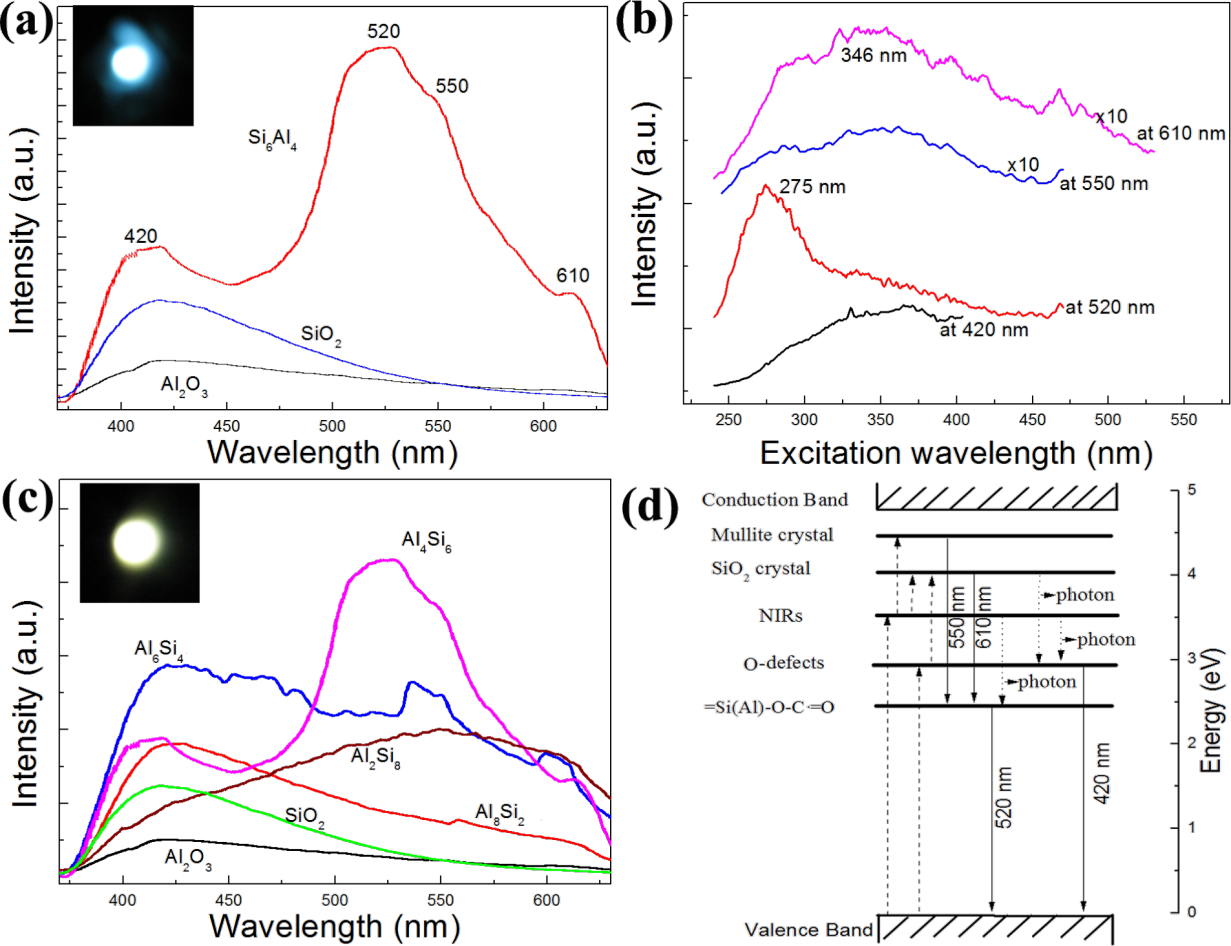
(a) PL comparison between the pure SiO_2_, Al_2_O_3_, and Al_4_Si_6_ nanofibers; (b) PLE spectra of Al_4_Si_6_ samples monitored at 420 nm, 520 nm, 550 nm, and 610 nm; (c) PL spectra of the obtained nanofibers with different Al/(Al–Si) ratios. The inset optical photos in (a) and (c) are the corresponding light emission spot of Al_6_Si_4_ and Al_4_Si_6_ samples, respectively. (d) Energy transfer diagram indicating the mechanism for Al_2_O_3_–SiO_2_ nanocomposite emission. The dashed lines represent light absorption, the solid lines radiative transitions, and the dotted lines nonradiative transitions.

We systematically studied the PL properties of the Al_2_O_3_–SiO_2_ composite nanofibers using a 325 nm He–Cd laser. Figure 5a compares the PL spectra of the pure Al_2_O_3_, SiO_2_, and Al_4_Si_6_ samples. It is noted here that the fluorescence spectrum of the Al_4_Si_6_ sample, which had the highest emission, can be separated into four components. One peak at 420 nm is due to oxygen-related defects (O defects) resulting from calcination of silica, alumina, or their composites [36], and another is a broad emission peak around 520 nm with a shoulder peak at 550 nm, which is the main contributor to the white emission. This 520 nm band can hardly be found in the pure SiO_2_ and Al_2_O_3_ samples, and this band is often assigned to radical carbonyl defects, ≡Si(Al)–O–C∙=O [16,37]. In addition, anther weak emission at approximately 610 nm is also an important contributor to the white emission.

To further investigate the absorption process of each PL band, we measured the PL excitation (PLE) spectra of Al_4_Si_6_ samples at various emission positions (420 nm, 2.95 eV; 520 nm, 2.38 eV; 550 nm, 2.25 eV; 610 nm, 2.03 eV), which are shown in Figure 5b. The PLE spectrum monitored at 420 nm is a broad absorbance band centered at around 346 nm, while the PLE spectrum monitored at 520 nm shows a 275 nm absorption peak (4.51 eV), together with a broad shoulder around 300–400 nm. According to the previous results, the 275 nm absorption peak might be attributed to the absorption of mullite components formed in the samples [38], and the broad band around 300–400 nm to the absorption by the near-interface regions between the SiO_2_ and mullite crystals [39]. Moreover, the 550 nm emission has a similar PLE spectrum to that of the 610 nm, indicating a similar origin of the light absorbance. From further comparison the energy of each band, it can be suggested that the 550 nm and 610 nm emissions are associated with the intersystem radiative crossing between mullites (or SiO_2_) and radical carbonyl defects (≡Si(Al)–O–C∙=O).

In order to further investigate the true origin of each band and how the Al- and Si-related components affect the PL behaviors of the composite nanofibers, we have measured the PL spectrum of the samples with different Al–Si ratios, as shown in Figure 5c. As for the PL behavior of Al_2_Si_8_ nanofibers, it exhibits a dark-yellow emission with a broad band centered at about 550 nm. It can be seen that the intensities of the 550 and 610 nm emissions are greatly enhanced while the 420 nm emission intensity is reduced as compared to that of pure SiO_2_. And the 550 and 610 nm emissions can be further enhanced with further addition of Al up to 40 mol %.

When the Al content increases to 60 mol %, the Al_6_Si_4_ nanofibers show a strong yellow-white emission, as demonstrated in the inset in Figure 5c. This result indicates that the 520 nm emission deceases with more Al content and less Si content, again suggesting that the 520 nm emission is associated with Si–Al defects (i.e., as the previous assignment of ≡Si(Al)–O–C∙=O). From the XRD results, it can be observed that the content of crystalline SiO_2_ decreases with the further increase of Al. At an Al content of 60 mol %, the samples exhibit a very high level of crystallized mullite. At the same time, less ≡Si(Al)–O–C∙=O defects can readily form during calcination in air.

Once excessive Al concentrations (80 and 100 mol %) are reached in the samples, the obtained nanofibers exhibit a dark-blue emission with the main emission peak at ≈420 nm. It can be seen that the 520, 550 and 610 nm luminescence bands almost disappear. From the XRD results, it can be seen that only the highest degree of crystallization of mullite is obtained, with very little SiO_2_ and ≡Si(Al)–O–C∙=O remaining in the samples. On the other hand, the 3A1_2_O_3_·2SiO_2_ (3:2) mullite components first increase with more Al addition; once excessive amounts of Al were added to the sample, the 2A1_2_O_3_∙1SiO_2_ (2:1) mullite components formed. It is know from previous work [38,40] that the 3:2 mullites possess a wide band gap in the range of 3.95–5.5 eV, which can be of benefit to the intersystem radiative crossing for ≡Si(Al)–O–C∙=O. However, the 2:1 mullites have a wide band gap of 7.7 ± 0.2 eV, which is too wide for our case.

Therefore, based on the above analysis, we assign an energy transfer mechanism to describe our PL results, as shown in Figure 5d. First, most of the energy needed for the excitation of radical carbonyl defects (≡Si(Al)–O–C∙=O) is absorbed by the near-interface region between the SiO_2_ and the mullites (absorption centered at 346 nm), and only a small proportion of the energy is by absorbed O defects. Next, a large part of the absorbed energy can be transferred nonradiatively to ≡Si(Al)–O–C∙=O (520 nm), while some energy can be easily transferred to an even higher energy band (SiO_2_ and mullite crystals), and rest to O defects (420 nm). At the same time, the energy transferred to mullite and SiO_2_ crystals can mainly intersystem radiatively cross to ≡Si(Al)–O–C∙=O, emitting weak light at 550 nm and 610 nm.

**Figure 6:**
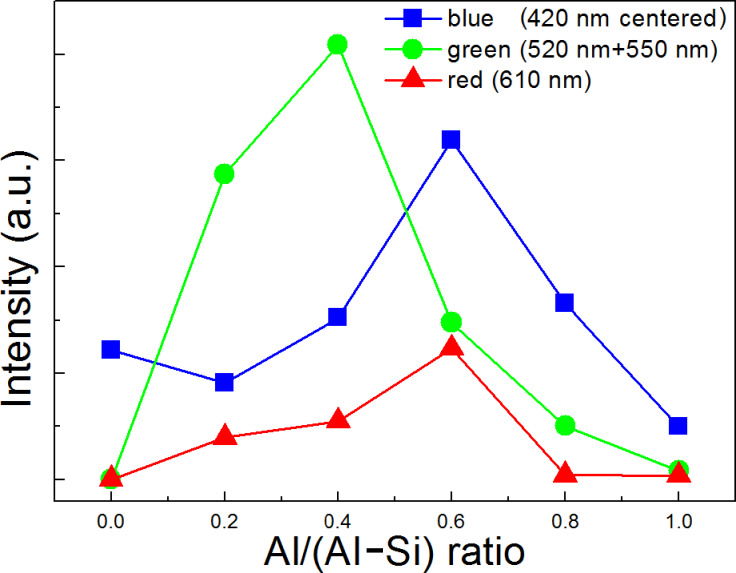
Integrated PL intensity of blue, green, and red luminescent centers as a function of Al/(Al–Si) ratios.

To better understand the effect of changing the Al/(Al–Si) ratio on the PL properties of the composite nanofibers, we analyzed the raw statistics of the PL intensity for each colored luminescent center, as shown in Figure 6. The intensity of each luminescent center was integrated over the intensity area, fitted using a Gaussian fit. The 420 nm-centered broad bands are regarded as blue light centers, the 520 nm and 550 nm bands are green light centers, and the 610 nm band as a red emission center. Obviously, a suitable dopant of Si or Al into the composite samples are required for light emission. It can be seen that the blue light centers, such as O defects (420 nm), first slightly decrease with the increasing Al/(Al–Si) ratio, and then increase, and reach their minimum value at an Al content of 60 mol % with further increase in Al content. This result indicates that suitable dopants of Si or Al are benefitial for this type of blue light emission. While both red and greeen centers first increase with increasing Al content (reaching their maximum value at Al contents of 0.6 and 0.4), they then decrease with further Al addition. Interestingly, at Al concentrations near 60 mol % and 40 mol %, the intensity ratio of the blue, green, and red emission components mimic white light better than the other samples’s emission, and output of blue-white and yellow PL was observed as shown in the PL spots in Figure 5a and Figure 5c.

## Conclusion

In summary, Al_2_O_3_–SiO_2_ composite nanofibers with different Al/(Al–Si) ratios were prepared by electrospinning in combination with post-calcination at 1200 °C in air. The obtained composite nanofibers are comprised of mullite and cristobalite nanocrystals, and the composite fibers with a Al/(Al–Si) ratio of approximately 60–80 mol % exhibited a coarse surface, due to the precipitation of mullite and cristobalite nanocrystals. Furthermore, PL experiments indicate that the white light emission can be tuned by the Al/(Al–Si) ratio. This is accomplished by tuning the intensity of each spectral component: violet-blue light from O defects, green emission from ≡(Si)Al–O–C∙=O, and red emission from the intersystem radiative crossing. This research may provide a new strategy for the preparation of environmentally friendly, white light luminescence materials.

## Experimental

### Preparation of Al_2_O_3_–SiO_2_ composite nanofibers

Poly(vinylpyrrolidone) (PVP, *M*_w_ ≈1,300,000) was purchased from Sigma-Aldrich, aluminum nitrate nanohydrate (Al(NO_3_)_3_·9H_2_O) and tetraethoxysilane (TEOS) were used for the Al and Si sources, respectively, both purchased from Shantou Chemical Corp., China. All other chemicals were purchased from Tianjin Chemical Company (Tianjin, China). All chemicals were analytically pure and used as received without any further purification.

Al_2_O_3_–SiO_2_ hetero-nanofibers were prepared by electrospinning, the details of which can be reviewed from previously published work [41–44]. Briefly, sol–gel aqueous solutions were prepared by dissolving TEOS, Al(NO_3_)_3_·9H_2_O, and PVP powder (10 wt %) in absolute ethanol. The mole ratios of Al/(Al–Si) were set at 0 mol %, 20 mol %, 40 mol %, 60 mol %, 80 mol %, and 100 mol %, and their corresponding samples are denoted as SiO_2_, Al_2_Si_8_, Al_4_Si_6_, Al_6_Si_4_, Al_8_Si_2_, and Al_2_O_3_, respectively. After strong magnetic stirring for 2 h, the mixture was transferred into a single-nozzle electrospinning setup. The voltage and distance applied between the needle tip and the collector was set as 10 kV and 15 cm, respectively. The as-spun fibers were collected on silicon or quartz substrates. After electrospinning, these as-spun Al_2_O_3_–SiO_2_ fibers were calcined in a tube furnace in air at 1200 °C for 2 h to obtain pure crystalline Al_2_O_3_–SiO_2_ nanofibers.

### Characterization

The crystalline structure, morphology and PL properties of the final products were investigated by X-ray diffraction (XRD, Philips X’pert Pro), field-emission scanning electron microscopy (FE-SEM, Hitachi S4800), transmission electron microscopy (TEM, JEM 3000F, JOEL), Fourier transform infrared spectroscopy (FTIR, IFS66v/S, 400–4000 cm^−1^), and fluorescence spectroscopy (RF-540, Shi-Madzu) using a 15 mW, 325 nm, He–Cd laser (spot size of about 1 mm) in addition to a spectroscopy with a FLS-920T spectroflorormeter (Edinburgh) with a 45 W, Xe lamp.
